# Nutrient composition, functional, and pasting properties of unripe cooking banana, pigeon pea, and sweetpotato flour blends

**DOI:** 10.1002/fsn3.455

**Published:** 2017-01-12

**Authors:** Ehimen R. Ohizua, Abiodun A. Adeola, Micheal A. Idowu, Olajide P. Sobukola, T. Adeniyi Afolabi, Raphael O. Ishola, Simeon O. Ayansina, Tolulope O. Oyekale, Ayorinde Falomo

**Affiliations:** ^1^ Department of Food Science and TechnologyFederal University of AgricultureAbeokutaOgun StateNigeria; ^2^ Food and Nutrition Research ProgrammeInstitute of Food SecurityEnvironmental Resources and Agricultural ResearchFederal University of AgricultureAbeokutaOgun StateNigeria; ^3^ Department of ChemistryFederal University of AgricultureAbeokutaOgun StateNigeria; ^4^ Department of Plant Protection and Crop ProductionFederal University of AgricultureAbeokutaOgun StateNigeria; ^5^ Department of Agricultural AdministrationFederal University of AgricultureAbeokutaOgun StateNigeria; ^6^ Food Security and Socio‐Economic Research ProgrammeFederal University of AgricultureAbeokutaOgun StateNigeria; ^7^ Laboratory ServicesInstitute of Food SecurityEnvironmental Resources and Agricultural ResearchFederal University of AgricultureAbeokutaOgun StateNigeria

**Keywords:** Functional properties, nutrient composition, pigeon pea, sweetpotato, unripe cooking banana

## Abstract

This study investigated some quality attributes of unripe cooking banana (UBF), pigeon pea (PPF), and sweetpotato (SPF) flour blends. Simplex centroid mixture design was used to obtain 17 blends from the flours. The nutrient composition, color, and functional properties of the blends were evaluated using standard methods. Data were subjected to analysis of variance and treatment means were compared using Duncan's multiple range test at 5% probability level. There were significant (*p* < .05) differences in the nutrient composition, and functional and pasting properties of the blends. The crude protein, crude fiber, ash, foaming capacity, emulsion capacity, and least gelation capacity of the blends increased as the PPF level increased. The blends had Na/K ratio of <1.0. The dispersibility, bulk density, water, and oil absorption capacities of the blends increased as SPF and UBF increased. The peak, setback, and final viscosities increased as UBF and SPF inclusion increased,whereas pasting temperature and time increased as the PPF level increased. The L*, a*, and b* values of the flour blends which were significantly (*p* < .05) different ranged from 79.58 to 102.71, −0.15 to 2.79, and 13.82 to 23.69, respectively. Cooking banana‐pigeon pea‐sweetpotato flour blends are desirable for alleviating malnutrition in Nigeria and developing new food formulations.

## 1 Introduction

According to Noorfarahzihah, Lee, Sharifudin, Mohd‐Fadzelly, and Hasmadi (2014), composite flour is defined as a mixture of flours from tubers (e.g. cassava, yam, sweetpotato) and/or legumes (e.g. soybean, pigeon pea, peanut) and/or cereal (e.g. maize, wheat, rice, millet, buckwheat). The use of composite flour has been identified by researchers as possible avenue of producing high‐quality nutritious food products and a means of reducing the huge amount of foreign exchange spent by Nigeria in the importation of wheat flour (Olaoye, Onilude, & Idowu, 2006; Nwosu, 2013; Vaughan, Afolami, Oyekale, & Ayegbokiki, 2014).

Basically, banana is an essential source of minerals (iron, zinc, selenium, magnesium, calcium, phosphorus, and potassium), vitamins (A, B1, B2, B6, and C), polyphenols, resistant starch, and antioxidants (Juarez‐Garcia, Agama‐Acevedo, Sa′Yago‐Ayerdi, Rodriguez‐Ambriz, & Bello‐Pe′rez, 2006; Vergara‐Valencia et al., 2007). Banana flour has been reported to increase the indigestible carbohydrates in food products and decreased glycemic response (Ovando‐Martinez, Sáyago‐Ayerdi, Agama‐Acevedo, Goñi, & Bello‐Pérez, 2009; Preedy, Watsoa, & Patel, 2011; Ayo‐Omogie & Ogunsakin, 2013; Osorio‐Díaz et al., 2014; Almanza‐Bentiez, Osorio‐Diaz, Mendez‐Montealvo, Islas‐Hernandez, & Bello‐Perez, 2015). Cooking banana, locally known as *ogede bello* was introduced into Nigeria by International Institute of Tropical Agriculture to check the incidence of Black Sigatoka disease. It was found to possess good agronomic characteristics and is less seasonal in production than dessert banana and plantain (Tshiunza, Lemchi, Onyeka, & Tenkouano, 2001; Adeniji, Tenkouano, Ezurike, Ariyo, & Vroh‐Bi, 2010). It is considered suitable for the preparation of flour because it is cheap, has high starch content and is less discolored during drying when compared with those prepared from dessert banana types (Suntharalingam & Ravindran, 1993). The use of cooking banana flour in some products such as complementary food and pasta has been reported (Ovando‐Martinez et al., 2009; Ayo‐Omogie & Ogunsakin, 2013; Osorio‐Díaz et al., 2014; Almanza‐Bentiez et al., 2015).

Pigeon pea (*Cajanus cajan*) is an important underutilized legume in south‐west Nigeria (Fasoyiro & Arowora, 2013), where it is locally known as *otili*. It is tolerant to drought and has wide adaptability to different environmental conditions (Troedson, Wallis, & Singh, 1990). It contains 20%–22% of all essential amino acids particularly lysine and 18%–35% protein, and therefore desirable in overcoming the incidence of protein‐energy malnutrition in Nigeria (Elegbede, 1998; Okpala & Okoli, 2011; Tiwari, Brennan, Jaganmohan, Surabi, & Alagusundaram, 2011; Anuonye, Jigam, & Ndaceko, 2012). Pigeon pea is rich in dietary minerals such as calcium, copper, phosphorus, magnesium, iron, sulfur, and potassium, and water‐ soluble vitamins such as thiamine, ascorbic acid, riboflavin, and niacin (Salunkhe, Chavan, & Kadam, 1986; Foodnet, 2002; Kaushal, Kumar, & Harma, 2012). It is a good source of slow‐release carbohydrates, making it a suitable raw material for the formulation of low glycemic index food product (Morales‐Medina, Mar Munio, Guadix, & Guadix, 2012). Trinidad, Mallillin, Loyola, Sagum, and Encabo (2010) and Srikaeo, Sukanya, and Sopade (2011) reported that composite flours from legumes (such as cowpea, pigeon pea) and unripe banana are good sources of dietary fiber, and could be used in the preparation of functional foods product. Consumption of high fiber food products has been linked to reduction in hermorrhoids and effective management of diabetes, high blood pressure, and obesity (Chukwu, Ezebuiro, Samuel, & Nwachukwu, 2013; Jaja & Yarhere, 2015).

Sweetpotato (*Ipomoea batatas*), a sweet‐tasting tuberous root, is ranked fourth in terms of consumption in the world after wheat, maize, and rice (Odebode, Egeonu, & Akoroda, 2008). Nigeria was ranked the leading producer of sweetpotato in Africa between 1993 and 2013 (Olatunde, Henshaw, Idowu, & Tomlins, 2016). It is rich in carbohydrate consisting mainly of starch and sugar (occurring as sucrose, glucose, and fructose), and small amounts of pectins, hemicellulose, and cellulose (Preedy et al., 2011; Saeed et al., 2012; Onabanjo & Ighere, 2014). Other chemical constituents of sweetpotato include protein, dietary fiber, β‐carotene, vitamins B, C, and E, and minerals such as manganese, potassium, and iron. It is a beneficial food for the diabetics, as preliminary studies on animals have revealed its ability to assist to stabilize blood sugar level and lower insulin resistance (Odebode et al., 2008; Preedy et al., 2011). Sweetpotato flour (SPF) is used for baking on its own or as composite flour, as well as a stabilizer in the ice‐cream industry. SPF is used as a dough conditioner in bread and biscuit manufacturing (Hagenimana, Carey, Gichuki, Oyunga, & Imungi, 1998; Shih, Adebowale, & Tafa, 2006; Hathorn, Biswas, Gichuhi, & Bovell‐Benjamin, 2008; Preedy et al., 2011).

The use of composite unripe cooking banana, pigeon pea, and sweetpotato flours in food commodities is expected to prevent and control certain metabolic diseases and improve the nutritional status of consumers (Annelisse, Susan, Frank, & William, 2011; Almanza‐Bentiez et al., 2015). The objective of this study was therefore to determine the nutrient composition, functional, and pasting properties of flour blends from unripe cooking banana, pigeon pea, and sweet potato.

## 2 Materials and Methods

### 2.1 Materials

Mature unripe cooking banana (*Musa cardaba* AAB) with colour index No. 1 (Osman & Abu‐Goukh, 2008) was purchased from a farmer in Ibadan, Oyo State. Sweetpotato (cream‐fleshed) was purchased from Osiele market in Abeokuta, Ogun State. Pigeon pea was purchased from Oshodi market in Oshodi, Lagos State.

### 2.2 Production of unripe banana flour

**Figure 1:**
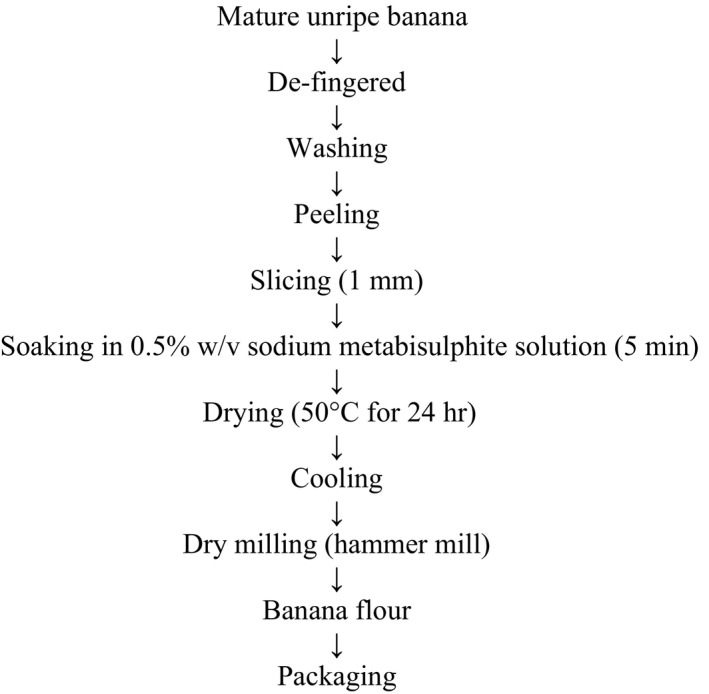
Preparation of banana flour

The procedure described by Daramola and Osanyinlusi (2006) in Figure 1 was adopted for the production of unripe cooking banana (UBF). The fruits were detached from the peduncle, defingered, washed, and peeled under water treated with 0.05% (w/v) sodium metabisulphite. The peeled banana fruits were then horizontally sliced with a kitchen cutter to an average thickness of 1 mm and allowed to remain in water containing 0.05% (w/v) sodium metabisulphite for 5 min to prevent browning of the resultant flour. The banana slices were then dried at 50°C for 24 hr in a Genlab Cabinet dryer (Model DC 500, Serial number 12B154). The dried chips were milled using Fritsch hammer mill (Serial number: 15.302/982) equipped with a 250 μm mesh sieve. The resultant pulp flour was packaged in polyethylene bag and stored at ambient temperature.

### 2.3 Production of pigeon pea flour

**Figure 2:**
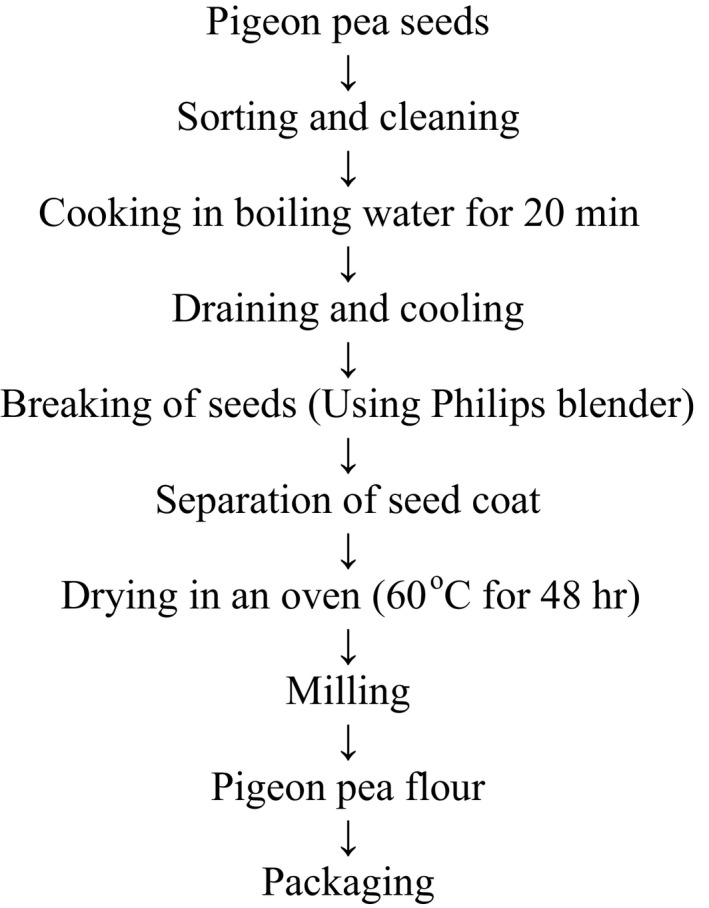
Preparation of pigeon pea flour

The method described by Fasoyiro et al. (2010) was modified in Figure 2 to produce Production of pigeon pea flour (PPF). Pigeon pea seeds were cleaned, sorted, and cooked in boiling water for 20 min. The seed coats were dehulled using a Philips blender, drained, and dried in the Genlab Cabinet dryer at 60°C for 48 hr. The dried pigeon pea seeds were allowed to cool at room temperature, and milled and packaged, as described for banana flour.

### 2.4 Production of sweetpotato flour

**Figure 3:**
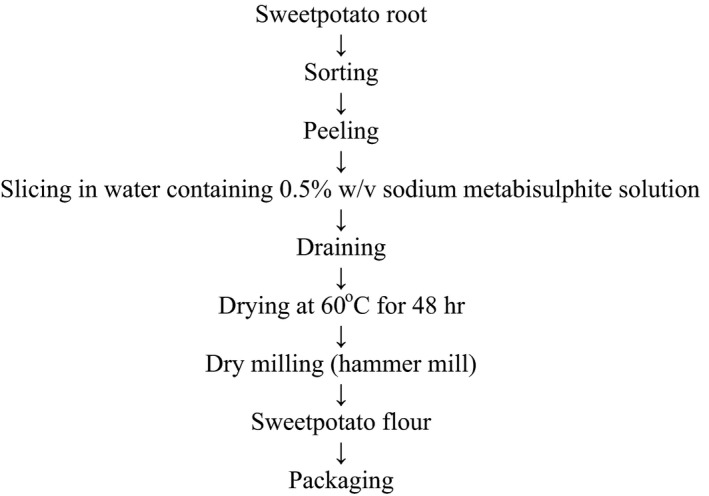
Preparation of pigeon sweetpotato flour

The method Figure 3 described by Onabanjo and Ighere (2014) was modified for the preparation of sweetpotato flour (SPF). The roots were sorted, cleaned with water to remove soils, peeled, and rewashed. The peeled roots were cut into chips and soaked in 0.05% (w/v) sodium metabisulfite for about 20 min, to prevent browning. The chips were drained, dried, milled, and packaged, as described for pigeon pea flour.

### 2.5 Blending of flour

Each flour was made to pass through 250 μm sieve (USA standard testing sieve; A.S.T.M.E.‐11 specification). Composite blends of UBF, PPF and SPF were mixed according to the different ratios generated from using the simplex centroid mixture design (Table 1). The blends were coded based on UBF:PPF:SPF as BYG (10:80:10), XZE (45:45:10), GLX (10:10:80), FTB (21.67:56.67:21.67), JER (45:10:45), KEW (10:45:45), AZE (10:10:80), PTE (33.3:33.3:33.3), REY (56.67:21.67:21.67), WIZ (10:80:10), QEB (80:10:10), BHZ (45:10:45), HEW (21.67:21.67:56.67), LEB (80:10:10), UBF (100:0:0), PPF (0:100:0), SPF (0:0:100)

**Table 1:** Composite flour blends obtained from unripe cooking banana, pigeon pea, and sweetpotato

Experimental run No/	Sample/formulation ID	Unripe banana flour X1	Pigeon pea flour X2	Sweetpotato Flour X3
1	BYG	10	80	10
2	XZE	45	45	10
3	GLX	10	10	80
4	FTB	21.67	56.67	21.67
5	JER	45	10	45
6	KEW	10	45	45
7	AZE	10	10	80
8	PTE	33.33	33.33	33.33
9	REY	56.67	21.67	21.67
10	WIZ	10	80	10
11	QEB	80	10	10
12	BHZ	45	10	45
13	HEW	21.67	21.67	56.67
14	LEB	80	10	10
15	UBF	100	0	0
16	PPF	0	100	0
17	SPF	0	0	100

The flours were thoroughly blended using a Kenwood mixer (Model HM 430) and packaged in different polyethylene bags for analyses.

### 2.6 Nutrient composition of flour blends

#### 2.6.1 Proximate composition and energy estimation

The composite flour blends were analyzed for their moisture, crude protein, crude fat, crude fiber, and ash contents according to the method described by AOAC (2010). Carbohydrate was determined by difference and energy content was determined by using the Atwater factor (carbohydrate and protein values were each multiplied by 4 kcal/g, whereas fat values were each multiplied by 9 kcal/g).

#### 2.6.2 Determination of vitamin C content

The titration method of AOAC (2010) was used to determine the vitamin C content of the flour blends. Two grams of each sample was macerated in 50 ml distilled water. To 50 ml of the prepared sample, equal volume of extraction solution (containing 15 g of phosphoric acid, 40 ml acetic acid, and up to 500 ml with distilled water) was added. Few drops of indicator (0.1 g thymol blue was dissolved in 10.75 ml of 0.02N sodium hydroxide solution and diluted to 250 ml water) was added to the aliquot and the resultant solution was titrated with Indophenol standard solution to obtain a rosy pink solution. Standard solution was prepared by dissolving 0.05 g ascorbic acid in 45 ml in the extraction solution and making up to 50 ml and titrated with indicator.

#### 2.6.3 Determination of total carotenoid

The total carotenoid content was determined by the method described by Chan and Cavaletto (1982) using UV/Visible spectrophotometer (Model: CE 2021 2000 series, serial no 923‐41) About 6 g of the sample was mixed with 5 g of hyflosupercel (celite, a filtration aid) and 15 ml of 70% methanol (v/v), and filtered through a Buchner funnel with filter paper. The residue was extracted two more times with 15 ml acetone‐petroleum ether 1:1 (v/v). The extracts were then transferred to 500 ml separating funnel. About 5 of 10% KOH in methanol (v/v) was added and the mixture allowed to stand for 1.5 h. Partition was achieved by adding 15 ml of petroleum ether and 20 ml of 20% NaCl (w/v), and mixing gently. The hypophasic (lower) layer was discarded. The epiphasic (upper) layer was washed three times with 20 ml of distilled water to remove excess acetone, filtered through a small funnel containing 3 g anhydrous sodium sulfate to remove residual water. The funnel was plugged with glass stopper to hold sodium sulfate. The filtrate was be made up to 100 ml with petroleum ether and the absorbance was measured at 450 nm; the wavelength of maximum absorption for β‐carotene in petroleum ether.

#### 2.6.4 Determination of mineral composition

The mineral content of the samples were determined using the wet method as described by Onwuka (2005). Calcium, iron, sodium, potassium, and magnesium element content were determined by Atomic Absorption Spectrophotometer (Model: Thermo scientific S series; Type: S4 AA System; NC: 942340030042; Model GE; Serial No: GE 712354, Thermo Electron Corporation, USA). One gram of each sample was weighed into a 125 ml Erlenmeyer flask and 20 ml of the acid mixture (containing 325 ml concentrated nitric acid, 40 ml perchloric acid, and 10 ml of sulfuric acid) was added. The content was mixed and heated gently in a digester (Buchi Digestion unit K‐424) at a medium heat under a fume hood and heating continued until dense white fume appeared. Heating continued for 30 s and then allowed to cool followed by the addition of 50 ml distilled water. The solutions were filtered using filter paper into a 100 ml volumetric flask and made up to mark with distilled water. The resultant solutions were read on the Atomic absorption Spectrophotometer. The instrument was calibrated with known standards (Standards for Mg: 0.5 ppm, 1.5 ppm, 3.0 ppm, 4.5 ppm. Standards for Ca: 5 ppm, 15 ppm, 30 ppm. Standards for K: 2,6,10 ppm Standards for Fe: 2,4,10 ppm; Standards for Mn: 2,5,10 ppm; Standards for Na: 2,4,6,10 ppm) and samples were analyzed at corresponding wavelength. The required hollow cathode lamp corresponding to the required mineral and holders in the lamp compartment was installed to determine concentration of each mineral. The dilution factor for magnesium was 10,000, whereas other mineral including calcium, iron, potassium, sodium, and manganese was 100.

#### 2.6.5 Determination of antinutritional factors

Tannin was determined according to the Folis—Denis colorimetric method as described by Jaffe (2003). The method described by Munro (2000) was used to determine the oxalate content, whereas a modified method of Ijarotimi and Babatunde (2013) was used for the determination of phytate and trypsin inhibitor contents.

### 2.7 Functional properties of flour blends

#### 2.7.1 Dispersibility

This was determined by the method described by Kulkarni, Kulkarni, and Ingle (1991). Ten grams of each sample was suspended in 200 ml measuring cylinder and distilled water was added to reach the 100 ml mark. The set‐up was stirred vigorously and allowed to settle for 3 hr. The volume of settled particles was recorded and subtracted from 100. The difference was reported as percentage dispersibility.

Dispersibility=100−volume of settled particle

#### 2.7.2 Bulk density

The bulk density (BD) of the sample was determined using the method described by Onwuka (2005). About 10 g of the sample was weighed into 50 ml graduated measuring cylinder. The sample was packed by gently tapping the cylinder on the bench top 10 times from a height of 5 cm. The volume of the sample was recorded.

$$\text{Bulk density(g/ ml)}=\frac{\text{Weight of sample}}{\text{Volume of sample after tapping}}$$

#### 2.7.3 Water absorption capacity

The method described by Onwuka (2005) was used. About 1 g of the flour sample was weighed into a 15 ml centrifuge tube and suspended in 10 ml of water. It was shaken on a platform tube rocker for 1 minute at room temperature. The sample was allowed to stand for 30 min and centrifuged at 1200 x g for 30 min. The volume of free water was read directly from the centrifuge tube.

$$\text{WAC}\left(\%\right)=\frac{\text{Amount of water added}-\text{Free water}}{\text{Weight of sample}}\times \text{density of water}\times 100$$

#### 2.7.4 Oil absorption capacity (OAC)

The method of Onwuka (2005) was used. One gram of the flour was mixed with 10 ml refined corn oil in a centrifuge tube and allowed to stand at room temperature (30 ± 2°C) for 1 hr. It was centrifuged at 1600 x g for 20 min. The volume of free oil was recorded and decanted. Fat absorption capacity was expressed as ml of oil bound by 100 g dried flour**.**

$$\left(\text{OAC}\%\right)=\frac{\text{Amount of oil added}-\text{Free oil}}{\text{Weight of sample}}\times \text{density of corn oil}\times 100$$

#### 2.7.5 Foaming capacity

Foaming capacity (FC) was determined according to the method described by Onwuka (2005). Two grams of flour sample was weighed and added to 50 ml distilled water in a 100 ml measuring cylinder, The suspension was mixed and properly shaken to foam and the total volume after 30 s was recorded. The percentage increase in volume after 30 s is expressed as foaming capacity.

$$\left(\text{Foaming capacity}\%\right)=\frac{\text{Volume after whipping}-\text{Volume before whipping}}{\text{Volume after whipping}}\times 100$$

#### 2.7.6 Emulsification capacity

Emulsification capacity (EC) was determined using the method of Kaushal et al. (2012). Two grams of the composite blend was blended with 25 ml distilled water at room temperature for 30 s. Thereafter, 10 ml of refined corn oil was added and the blending continued for another 30 s before transferring into a centrifuging tube. Centrifugation was done at 640 x g for 5 min. The volume of oil separated from the sample after centrifuging was read directly from the tube. Emulsification capacity was expressed as the amount of oil emulsified and held per gram of sample.

$$\left(\text{Emulsification capacity}\%\right)=\frac{\text{Height of emulsified layer}}{\text{Height of whole solution in the centifuge tube}}\times 100$$

#### 2.7.7 Least gelation concentration

Least gelation concentration (LGC) of flours was determined by the method of Sathe, Desphande, and Salunkhe (1982). Sample suspensions of 2%–20% (w/w) for each composite flour blend were prepared in distilled water and the dispersion was transferred into a test tube. It was heated in boiling water bath for 1 hr and rapidly cooled in a bath of cold water. The test tubes were further cooled at 4°C for 2 hr. The least gelation concentration is the concentration the sample did not fall down or slip when the test tube was inverted.

### 2.8 Determination of pasting properties of flour blends

This was determined using the Rapid Visco Analyser (RVA TECMASTER, Perten Instrument) as described by Newport Scientific (1998). The sample was turned into slurry by mixing 3 g of the sample with 25 ml of water inside the RVA can. The can was inserted into the tower, which was then lowered into the system. The slurry was heated from 50°C to 95°C and cooled back to 50°C within 14 min. Parameters estimated were peak, trough, final, breakdown and setback viscosities, pasting temperature, and time to reach peak viscosity.

### 2.9 Determination of flour blends’ color

The color intensity of the composite flour blends were measured using a Konica Minolta Colour Measuring System (Chroma meter CR‐410, Minolta LTD Japan) as described by Ahmed and Hussein (2014).

### 2.10 Data analysis

The data collected from the proximate, functional, antinutritional analysis, and pasting properties were presented as means of three determinations. The data were subjected to one‐way analysis of variance using SPSS statistical software version 21.0. The mean were separated by applying Duncan multiple Range test at 95% confidence level (*p* < .05).

## 3 Results and Discussion

### 3.1 Proximate composition and energy content

The Proximate composition and energy content of the blends is presented in Table 2. The moisture content ranged from 8.51% in PPF to 11.31% in LEB. Moisture content is an important parameter in flour which significantly affects shelf life of food product. Flour products with moisture content less than 13% are more stable from moisture‐dependent deterioration (Shahzadi, Butt, Rehman, & Sharif, 2005). The protein content was between 2.89 and 25.92%. The protein content increased as the percentage inclusion of PPF. This justifies the need for the fortification of SPF and UBF with this legume. The crude fat content was between 0.50% and 2.34% in AZE and UBF, respectively. Fat content usually plays a role in the shelf life stability of flour samples. The relatively low fat content of the composite blends makes them suitable raw materials in the formulation of a variety of food products for the elderly. The crude fiber content was between 0.75% in BYG and 2.97% in UBF. The fiber content increased as the percentage inclusion of PPF and UBF increased. This shows that the composite blends are good sources of fiber and can be used in the preparation of functional food products. Consumption of high fiber food products has been linked to reduction in hermorrhoids, diabetes, high blood pressure, and obesity (Chukwu et al., 2013; Jaja & Yarhere, 2015). The ash content ranged from 0.51% to 3.18% in REY and BYG, respectively. The total ash content of the blends increased as the inclusion level of SPF and PPF increased. The carbohydrate content which ranged from 59.29% (PPF) to 82.49% (JER) increased as the percentage inclusion of UBF and SPF increased, may be because these flours contain high amount of starch and sugar content (Saeed et al., 2012; Ayo‐Omogie & Ogunsakin, 2013). The energy content was between 346.89 kcal/g and 372.75 kcal/g. This high‐energy content of the composite flour may be advantageous for formulation of breakfast cereal and complementary foods (Iwe, Van Zauilichem, Nggody, & Ariahu, 2001). Carotenoid which is a precursor of vitamin A ranged from 26.00 μg/100 g in PTE to 174 μg/100 g in KEW. The carotenoids content increased as percentage inclusion of SPF increased. However, carotenoid content of the flour blends is considered low. This may be due to the variety of sweetpotato used and poor post harvest handling. Orange‐fleshed sweetpotato roots have been reported (Mills et al., 2015) to possess a higher amount of carotenoids than the cream‐fleshed sweetpotato root used in this study. Hagenimana et al. (1998) and Mills et al. (2015) also reported a loss in carotenoid in SPF during processing and improper storage condition as a result of oxidation. The presence of vitamin C signifies that the processing method adopted was able to retain small amount of this nutrient. Vitamin C is soluble in water, much of it is lost when food materials are washed slowly, soaked or boiled and the cooking water discarded, oxidized especially in an alkaline medium and on exposure to heat and light traces of metals particularly copper and iron.

**Table 2:** Nutrient composition and energy content of composite flour blends obtained from unripe cooking banana, pigeon pea, and sweetpotato

UBF:PPF:SPF code	Moisture content (%)	Crude protein (%)	Crude fat (%)	Crude fiber (%)	Total ash (%)	Carbohydrate content (%)	Energy value (kcal/g)	Total carotenoids (μg/100 g)	Vitamin C content (μg/100 g)
10:80:10	10.51^f^	17.01^p^	1.43 ^h^	0.75^a^	3.18^p^	67.15^b^	349.42^a^	92.00^d^	0.21^bc^
45:45:10	10.51^f^	13.41^n^	1.28^g^	1.11^c^	2.34^k^	71.36^d^	372.75^b^	139.00^b^	0.17^abc^
10:10:80	9.43^b^	4.13^d^	0.70^d^	2.09^gh^	1.33^d^	82.34^jk^	352.10^a^	130.00^f^	0.17^abc^
21.67:56.67:21.67	9.81^c^	5.22^g^	1.66^j^	2.67^j^	2.65^m^	77.99^f^	347.80^a^	157.00^g^	0.19^abc^
45:10:45	10.05^cde^	4.51^e^	0.63^bc^	1.01^bc^	1.33^d^	82.49^k^	353.60^a^	152.00^c^	0.19^abc^
10:45:45	10.51^f^	12.83^m^	2.01^l^	2.23^i^	3.06	69.38^c^	346.87^a^	174.00^e^	0.14^a^
10:10:80	10.31^ef^	3.91^c^	0.51^a^	1.39^d^	1.67^g^	82.24^jk^	349.10^a^	130.00^f^	0.21^bc^
33.3:33.3:33.3	10.51^f^	9.87^l^	1.56^i^	0.99^b^	1.76^i^	75.33^e^	354.79^a^	26.00^a^	0.19^abc^
56.67:21.67:21.67	9.91^cd^	7.41^j^	0.81^d^	1.08^bc^	0.51^a^	80.29^i^	358.08^ab^	39.00^b^	0.17^abc^
10:80:10	10.51^f^	16.16	1.50^hi^	1.87^f^	3.04^n^	66.94^b^	345.85^a^	92.00^d^	0.15^ab^
80:10:10	11.31^g^	9.38^k^	1.04^f^	1.58^e^	1.69^h^	75.01^e^	346.89^a^	39.00^b^	0.17^abc^
45:10:45	10.11^cde^	4.79^f^	0.66^c^	1.07^bc^	1.41^e^	81.97^j^	352.94^a^	54.00^c^	0.21^bc^
21.67:21.67:56:67	9.90^cd^	6.88^h^	0.91^e^	2.03^g^	1.86^j^	78.42^g^	349.39^a^	39.00^b^	0.21^bc^
80:10:10	11.31^g^	7.19^i^	0.57^ab^	0.79^a^	1.49^f^	78.64^g^	348.53^a^	39.00^b^	0.23^c^
100:0:0	10.20^def^	3.47^b^	2.34^m^	2.96^l^	1.09^b^	79.93^h^	354.68^a^	ND	0.27^c^
0:100:0	8.51^a^	25.92^q^	1.78^k^	2.14^hi^	2.37^l^	59.29^a^	356.83^ab^	ND	0.19^abc^
0:0:100	10.00^cde^	2.89^a^	1.12^f^	2.78^k^	1.17^c^	82.04^j^	349.82^a^	ND	0.25^c^

Mean values with different superscripts within a column are significantly different (*p* < .05).

UBF, Unripe banana flour; PPF, Pigeon pea flour; SPF, Sweetpotato flour.

### 3.2 Mineral composition

There was a significant difference (*p* < .05) in the mineral content of the composite flour blends with respect to magnesium, sodium, calcium, and potassium (Table 3). It ranged from 70.89 to 753.60 mg/100 g for magnesium, 2.51 to 11.67 mg/100 g for sodium, 0.15 to 1.26 mg/100 g for iron, 1.65 to 18.40 mg/100 g for potassium, and 1.10 to 7.34 mg/100 g for calcium. Manganese was not detected in the flour blends. Minerals are essential for the maintenance of the overall mental physical wellbeing and are important constituents for the development and maintenance of bones, teeth, tissues, muscles, blood, and nerve cells. They aid acid base balance, response of the nerves to physiological stimulation and blood clotting (Wardkaw & Kessel, 2002).

**Table 3:** Mineral Composition of composite flour blends obtained from unripe banana, pigeon pea, and sweet potato flours

UBF:PPF:SPF	Magnesium (mg/100 g)	Sodium (mg/100 g)	Iron (mg/100 g)	Potassium (mg/100 g)	Calcium (mg/100 g)
10:80:10	738.18^p^	5.39^f^	0.15^a^	3.22^i^	5.83^m^
45:45:10	729.47^m^	4.64^e^	0.16^b^	3.83^j^	3.26^e^
10:10:80	620.72^d^	10.18^m^	0.22^d^	1.67^b^	3.57^f^
21.67:56.67:21.67	641.92^f^	9.18^l^	0.22^d^	1.92^c^	3.78^g^
45:10:45	683.60^j^	7.95^j^	0.19^c^	2.17^e^	1.81^b^
10:45:45	667.59^g^	8.73^k^	0.17^b^	2.01^d^	4.35^j^
10:10:80	627.07^e^	10.28^n^	0.19^c^	1.65^a^	2.59^c^
33.3:33.3:33.3	681.24^i^	7.12^i^	0.21^d^	2.49^f^	3.03^d^
56.67:21.67:21.67	753.60^q^	5.64^g^	0.19^c^	3.14^h^	4.74^k^
10:80:10	730.73^o^	5.39^f^	0.15^a^	3.22^i^	5.75^l^
80:10:10	727.80^l^	3.87^c^	0.20^d^	4.69^l^	1.13^a^
45:10:45	676.79^h^	7.95^j^	0.19^c^	2.17^e^	1.90^b^
21.67:21.67:56:67	702.90^k^	6.08^h^	0.17^b^	2.87^g^	3.80^h^
80:10:10	727.85^m^	3.85^b^	0.19^c^	4.59^k^	1.10^a^
100:0:0	78.02^b^	2.51^a^	1.26^h^	18.26^n^	4.13^i^
0:100:0	81.29^c^	4.58^d^	0.95^g^	17.43^m^	7.34^n^
0:0:100	70.89^a^	11.67^o^	0.41^f^	18.40^o^	3.87^h^

Mean values with different superscripts within a column are significantly different (*p* < .05).

UBF, Unripe banana flour; PPF, Pigeon pea flour; SPF, Sweetpotato flour.

### 3.3 Antinutrient content

Significant (*p* < .05) difference existed for tannin, oxalate, phytate, and tryspin inhibitor contents of the composite flour blends as shown in Table 4. The tannin values which ranged from 32.63 mg/100 g in BHZ to 1020.64 mg/100 g in XZE increased as percentage of UBF and PPF increased. Tannins are polyhydric phenols present in virtually all parts of plants and are known to inhibit trypsin, chymotrypsin, amylase, and lipase activities (Inyang & Ekop, 2015). The amount of tannin in the blends may provoke an astringent reaction in the mouth, decrease palatability of food, cause damage to intestinal tract, and enhance carcinogenesis (Onwuka, 2005).

**Table 4:** Antinutritional content of composite flour blends obtained from unripe cooking banana, pigeon pea, and sweetpotato

UBF:PPF:SPF	Tannin (mg/100 g)	Oxalate mg/100 g	Phytate mg/100 g	Tryspin inhibitor (mg/100 g)
10:80:10	208.25^m^	1.26^h^	1988.61^p^	199.63^o^
45:45:10	1020.64^q^	0.94^c^	2257.84^q^	210.85^p^
10:10:80	52.74^h^	1.75^o^	569.30^b^	96.77^d^
21.67:56.67:21.67	449.53^p^	1.32^i^	1166.63^k^	197.11^m^
45:10:45	32.93^b^	1.08^e^	616.94^d^	126.95^k^
10:45:45	271.60^n^	1.43^k^	1351.00^n^	145.69^l^
10:10:80	52.44^g^	1.72^n^	569.00^a^	96.47^c^
33.3:33.3:33.3	447.46^o^	1.37^j^	1214.48^l^	93.39^b^
56.67:21.67:21.67	44.33^e^	1.12^f^	903.53^i^	121.84^e^
10:80:10	207.95^l^	1.23^g^	1988.31^o^	199.33^n^
80:10:10	38.45^c^	0.78^a^	753.95^f^	121.87^f^
45:10:45	32.63^a^	1.05^d^	616.64^c^	126.65^j^
21.67:21.67:56:67	130.39^j^	1.62^m^	1033.41^j^	125.78^h^
80:10:10	38.75^d^	0.81^b^	754.25^g^	122.17^g^
100:0:0	51.84^f^	1.54^l^	638.25^e^	82.46^a^
0:100:0	144.84^k^	1.08^e^	853.40^h^	125.86^i^
0:0:100	67.42^i^	1.42^k^	1263.58^m^	281.01^q^

Mean values with different superscripts within a column are significantly different (*p* < .05).

UBF, Unripe banana flour; PPF, Pigeon pea flour; SPF, Sweetpotato flour.

The oxalate content which ranged from 8.12 mg/100 g to 17.47 mg/100 g increased as the percentage inclusion of SPF and UBF increased. Sweetpotato and unripe banana have been associated with high amount of oxalate (Onwuka, 2005).

The phytate content which varied from 569.00 mg/100 mg in AZE to 2257.84 mg/100 g in XZE increased as the inclusion of SPF and PPF increased. Pigeon pea and sweetpotato are rich in phytate and presence of this compound may reduce the bioavailability of minerals such as iron, magnesium, calcium in the flour blends.

The tryspin inhibitor which ranged from 93.39 mg/100 g to 210.85 mg/100 g, increased as the percentage inclusion of UBF and PPF increased. The level of trypsin inhibitor may hamper protein digestibility, however, it has been reported that trypspin inhibitor are thermoliable and may be destroyed with the application of heat.

### 3.4 Functional properties

Functional properties of a food material are parameters that determine its application and end use (Adeleke & Odedeji, 2010). It usually shows how the food materials under investigation will interact with other food components directly or indirectly affecting processing applications, food quality, and ultimate acceptance. Significant (*p* < .05) differences were seen in some functional properties of the composite flour blends (Table 5). Dispersibility which ranged from 52.0% to 79.0% increased as PPF and UBF level increased. Dispersibility is an index that measures how well flour or flour blends can be rehydrated with water (Kulkarni et al., 1991). All the flour blends have relatively high dispersibility signifying that they will reconstitute easily to fine consistent dough or pudding during mixing (Adebowale, Sanni, & Ladapo, 2008).

**Table 5:** Functional properties of composite flour blends obtained from unripe cooking banana, pigeon pea, and sweetpotato

UBF:PPF:SPF	Dispersibility%	BD (g/ml)	WAC%	OAC %	FC%	EC %	LGC %
10:80:10	66.00^de^	0.76^d^	239.04^abc^	95.83^ab^	12.88^h^	61.19^de^	14^f^
45:45:10	66.00^de^	0.83^e^	298.61^cd^	95.65^ab^	8.96^def^	51.22^b^	10^d^
10:10:80	66.00^de^	0.92^g^	226.30^ab^	98.65^ab^	3.95^c^	63.02^e^	10^d^
21.67:56.67:21.67	68.00^ef^	0.83^e^	271.32^bcd^	103.42^ab^	10.00^fg^	56.25^bcd^	10^d^
45:10:45	69.00^ef^	0.72^c^	278.87^bcd^	95.62^ab^	7.88^de^	61.91^de^	10^d^
10:45:45	68.00^ef^	0.92^g^	303.22^cd^	123.67^bcd^	3.99^bc^	54.20^bc^	8^c^
10:10:80	79.00^g^	0.88^f^	226.32^ab^	92.63^a^	3.00^ab^	63.02^e^	10^d^
33.3:33.3:33.3	60.00^c^	0.76^d^	199.60^a^	92.66^a^	7.43^b^	61.99^de^	8^c^
56.67:21.67:21.67	65.00^d^	0.82^e^	256.09^abc^	154.03^e^	8.42d^ef^	59.58^cde^	8^c^
10:80:10	66.00^de^	0.76^d^	239.26^abc^	95.79^ab^	11.76^gh^	61.19^de^	14^f^
80:10:10	65.00^d^	0.82^e^	252.13^abc^	95.42^ab^	9.80^ef^	52.38^b^	8^c^
45:10:45	60.00^c^	0.72^c^	278.89^bcd^	95.32^ab^	7.88^de^	61.91^de^	8^c^
21.67:21.67:56:67	71.00^fg^	0.71^c^	258.83^abc^	96.01^ab^	7.43^d^	64.67^e^	10^d^
80:10:10	65.00^d^	0.89^f^	252.13^abc^	95.61^ab^	9.31^def^	52.38^b^	8^c^
100:0:0	52.00^a^	0.66^b^	299.31^cd^	131.79^cde^	12.78^h^	20.68^a^	6^b^
0:100:0	64.00^d^	0.48^a^	302.11^cd^	111 .06^abc^	12.08^h^	61.22^de^	12^e^
0:0:100	56.00^b^	0.77^d^	336.58^d^	141.75^de^	2.01^a^	25.80^a^	4^a^

Mean values with different superscripts within a column are significantly different (*p* < .05).

UBF, Unripe banana flour; PPF, Pigeon pea flour; SPF, Sweetpotato flour; EC, Emulsification capacity; LGC, Least gelation concentration.

The result of bulk density (BD) is used to evaluate the flour heaviness, handling requirement and the type of packaging materials suitable for storage and transportation of food materials (Oppong, Arthur, Kwadwo, Badu, & Sakyi, 2015). The bulk density which varied from 0.48 g/ml to 0.92 g/ml increased as the incorporation level of UBF and SPF increased. It was observed that the composite flour blends are heavy so a lower amount of the flour may be packaged within a constant volume (Oppong et al., 2015).

The water absorption capacity ranged from 199.60% to 336.58%. The ability of the composite blend to absorb water increased as the level of incorporation of UBF and SPF increased. This may be attributed to the low protein and high carbohydrate contents of SPF and UBF, as carbohydrates have been reported to greatly influence the water absorption capacity (WAC) of foods (Anthony et al., 2014). From the results, all the composite blends showed favorable WAC thus making them suitable raw material or functional ingredients in the development of ready‐to‐eat food products, soups, gravies, and baked products.

Oil absorption capacity (OAC) measures the ability of food material to absorb oil. OAC varied from 92.93% to 154.03% showing the flour blend has high oil absorption capacity as a result of the hydrophobic character of protein in the flour. The presence of protein exposes more non‐polar amino acids to the fat and enhances hydrophobicity as a result of which the flour absorbs more oil (Oluwalana, Oluwamukomi, Fagbemi, & Oluwafemi, 2011).

EC measures the maximum amount of oil emulsified by protein in a given amount of flour. EC which varied ranged from 25.80% to 64.67% increased as the level of inclusion of PPF increased. Sathe and Diphase (1981) reported that high emulsification capacity may be due to the globular nature of the major protein.

Foaming capacity (FC) is used to determine the ability of the flour to foam which is dependent on the presence of the flexible protein molecules which decrease the surface tension of water (Asif‐Ul‐Alam, Islam, Hoque, & Monalis, 2014). The values for foaming capacity which ranged from 2.01% to 12.88% decreased as the percentage inclusion of PPF decreased. This was expected since the protein content of PPF is considerably higher than UBF and SPF. Similar results (23.5%–65.0%) were reported by Kiin‐Kabari, Eke‐Ejiofor, and Giami (2015) as the substitution of bambara groundnut increased in wheat/plantain flours.

Least gelation capacity (LGC) measures the minimum amount of flour needed to form a gel in a measured volume of water. It varies from flour to flour depending on the relative ratios of their structural constituents like protein, carbohydrates, and lipids (Abbey & Ibeh, 1988). LGC which varied from 4% to 14% increased as percentage inclusion of PPF increased. The increasing concentration of protein enhances the interaction among the binding forces which in turn increases the gelling ability of the flour (Lawal, 2004). It was observed that the higher the LGC, the higher the quantity of flour needed to form a gel and the lower the LGC the better the gelling ability of the flour.

### 3.5 Pasting properties of composite flour blends

The pasting characteristics of the composite flour blends as shown in Table 6 were significantly (*p* < .05) different. Peak viscosity which is the maximum viscosity developed during or soon after the heating aspect of the test (Adebowale et al., 2008) ranged from 4.17 to 413.04 RVU, and decreased with increased PPF inclusion. The low peak viscosity seen in some composite blends indicates that the flour blends without modifications may be suitable for the preparation of complementary foods.

**Table 6:** Pasting properties of composite flour blends obtained from unripe cooking banana, pigeon pea, and sweetpotato

UBF:PPF:SPF	Peak viscosity (RVU)	Trough viscosity (RVU)	Breakdown viscosity (RVU)	Final viscosity (RVU)	Setback viscosity (RVU)	Pasting time (minutes)	Pasting temperature (^o^C)
10:80:10	70.42^b^	58.25^bc^	3.17^a^	78.33^abc^	24.92^ab^	7.00^d^	89.58^d^
45:45:10	129.71^c^	17.71^ab^	145.38^f^	35.25^ab^	35.25^bc^	5.37^bc^	81.93^bc^
10:10:80	271.00^ef^	180.58^fg^	90.42^e^	247.88^efg^	67.29^de^	4.50^b^	81.63^b^
21.67:56.67:21.67	111.25^c^	101.58^de^	2.58^a^	133.42^bcd^	22.88^ab^	6.18^cd^	83.00^c^
45:10:45	283.21^f^	225.58^ghij^	99.29^e^	295.29^fg^	111.38^g^	4.61^b^	80.73^ab^
10:45:45	135.25^c^	118.92^e^	16.33^ab^	157.38^cde^	38.46^bc^	5.40^bc^	81.10^ab^
10:10:80	289.50^f^	182.17^fg^	107.29^e^	251.50^efg^	69.34^de^	4.40^b^	80.74^ab^
33.3:33.3:33.3	202.92^d^	170.88^f^	32.79^bc^	225.88^def^	55.00^cd^	5.24^bc^	81.88^bc^
56.67:21.67:21.67	273.29^ef^	214.20^fghi^	59.08^d^	159.46^cde^	65.96^de^	5.10^bc^	81.95^bc^
10:80:10	66.29^b^	61.75^cd^	4.54^a^	88.79^abc^	27.04^b^	7.00^d^	89.58^d^
80:10:10	329.50^g^	233.79^hij^	95.71^e^	331.69^fg^	97.83^fg^	4.80^b^	81.13^ab^
45:10:45	295.16^f^	208.00^fgh^	87.17^e^	290.41^fg^	82.42^ef^	4.73^b^	80.75^ab^
21.67:21.67:56:67	246.75^e^	190.75^fgh^	56.00^cd^	257.71^efg^	66.96^de^	5.00^b^	81.20^ab^
80:10:10	348.92^g^	263.96^j^	84.96^e^	353.67^g^	89.71^efg^	4.90^b^	81.10^ab^
100:0:0	413.04^h^	254.96^ij^	158.08^f^	343.34^g^	88.38^efg^	4.74^b^	79.85^a^
0:100:0	4.17^a^	3.92^a^	0.25^a^	6.00^a^	2.08^a^	6.73^d^	90.89^d^
0:0:100	409.41^h^	213.00^fghi^	196.54^g^	268.67^fg^	55.67^cd^	3.09^a^	80.95^ab^

Mean values with different superscripts within a column are significantly different (*p* < .05).

UBF, Unripe banana flour; PPF, Pigeon pea flour; SPF, Sweetpotato flour.

**Table 7:** Color intensity of composite flour blends obtained from unripe cooking banana, pigeon pea, and sweetpotato

UBF:PPF:SPF	L*	a*	b*
10:80:10	86.99^fg^	−0.22^b^	15.06^bcd^
45:45:10	81.99^abc^	1.49^fg^	15.53^de^
10:10:80	86.62^efg^	0.71^c^	15.29^cd^
21.67:56.67:21.67	84.78^def^	0.72^c^	15.45^de^
45:10:45	81.78^ab^	1.67^g^	14.58^abcd^
10:45:45	86.84^efg^	0.37^b^	14.79^abcd^
10:10:80	84.38^cde^	1.12^de^	16.58^e^
33.3:33.3:33.3	85.01^def^	0.88^cd^	14.58^abcd^
56.67:21.67:21.67	83.39^bc^	1.37^ef^	14.14^abc^
10:80:10	88.72^g^	−0.15^a^	16.55^e^
80:10:10	82.13^bc^	2.21^1^	13.90^ab^
45:10:45	81.78^ab^	1.67^g^	14.58^abcd^
21.67:21.67:56:67	86.57^efg^	0.83^c^	15.50^de^
80:10:10	79.58^a^	2.25^i^	14.57^abcd^
100:0:0	89.42^g^	2.79^j^	16.64^e^
0:100:0	88.05^g^	1.91^h^	23.69^g^
0:0:100	102.71^h^	1.52^fg^	19.87^f^

Mean values with different superscripts within a column are significantly different (*p* < .05).

UBF, Unripe banana flour; PPF, Pigeon pea flour; SPF, Sweetpotato flour.

Trough viscosity measures the ability of the paste or gel formed to withstand breakdown during cooling. (Ayo‐Omogie & Ogunsakin, 2013). Trough viscosity which ranged from 17.71 to 263.96 RVU increased as the percentage inclusion level of SPF and UBF increased. This may be due to the swelling capacity of the starch granules in SPF and UBF.

Breakdown viscosity measures the ability of the flour to withstand heating and shear stress during cooking (Adebowale, Sanni, & Awonorin, 2005). The breakdown viscosity ranged from 0.25 RVU to 158.08 RVU.

Final viscosity (FV) measures the ability of the starch to form starch and viscous paste or gel after cooking and cooling (Maziya‐Dixon, Dixon, & Adebowale, 2007). FV which ranged from 6.00 to 353.67 RVU increased as the percentage inclusion of SPF and UBF increased. This may attributed to high carbohydrate content in both flours.

Setback viscosity (SV) gives an idea about retrogradation tendency of starch in flour sample. The SV which ranged from 2.08 to 111.38 RVU increased as the percentage inclusion of PPF reduced. This indicates reduction in the textural characteristics of the samples since setback has been correlated with texture (Adebowale et al., 2005).

Peak time is the time at which the peak viscosity occurred in minutes and it is a measure of the cooking time of the flour (Adebowale et al., 2005). Peak time which ranged from 4.40 min to 7.00 min increased as the inclusion of PPF increased.

Pasting temperature is the temperature at which the first detectable increase in viscosity is measured and it is an index characterized by the initial change due to swelling of starch (Julanti, Rusmarilin, & Ridwansyah, 2015). A high pasting temperature usually indicates the flour has high water absorption capacity (Julanti et al., 2015). The pasting temperature ranged from 79.85°C to 90.89°C increased as the percentage PPF level increased.

### 3.6 Colour of composite flour blends

Color values (L*, a*, b*) of the different composite blends were significantly (*p* < .05) different (Table 6). The L* values which ranged from 79.58 to 102.71 increased with increase inclusion of UBF and SPF. The a* and b* values of different flours varied between −0.15 to 2.79 and 13.82 to 23.69, respectively. The a* values were positive, thus indicating the predominance of red color over green color except for sample BYG and WIZ. The b* value which shows yellowness of the flour increased as PPF and SPF increased. All the flour blends had positive b* values, indicating a strong predominance of the yellow coloration, over blue.

## 4 Conclusion

The nutrient composition, functional, and pasting properties of unripe cooking banana, pigeon pea, and sweetpotato flour blends was studied. The crude protein, crude fiber, ash, foaming capacity, emulsion capacity, and least gelation capacity of the blends increased as the PPF level increased. The dispersibility, bulk density, water, and oil absorption capacities of the blends increased as SPF and UBF increased. The blends were rich in magnesium and had Na/K ratio of <1.0. The peak, final, setback, and final viscosities increased as UBF and SPF inclusion increased while pasting temperature and time of the composite blends increased as the percentage PPF level increased. The color of the blends was light and showed a predominance of yellow color. Furthermore, the other quality attributes of the blends showed that they could be used in the preparation of complementary foods and as substitute raw materials for wheat in the production of pastas, puddings and biscuits. The study paves way for enhanced utilization of cooking banana, pigeon pea, and sweetpotato in the country. This paper revealed that cooking banana, pigeon pea, and sweet potato flour blends are good sources of protein, fiber, and carotenoids and are desirable to improve the nutritional wellbeing of Nigerians.

## Conflict of Interest

None declared.
